# The Brominated Compound Aeroplysinin-1 Inhibits Proliferation and the Expression of Key Pro- Inflammatory Molecules in Human Endothelial and Monocyte Cells

**DOI:** 10.1371/journal.pone.0055203

**Published:** 2013-01-28

**Authors:** Beatriz Martínez-Poveda, Javier A. García-Vilas, Casimiro Cárdenas, Esther Melgarejo, Ana R. Quesada, Miguel A. Medina

**Affiliations:** 1 Department of Molecular Biology and Biochemistry, Faculty of Sciences, University of Málaga, Málaga, Spain; 2 CIBER de Enfermedades Raras (CIBERER), Málaga, Spain; Center for Cancer Research, National Cancer Institute, United States of America

## Abstract

Aeroplysinin-1 is a brominated antibiotic used by some sponges for defense against bacterial pathogen invasion. Aeroplysinin-1 has a wide spectrum of anti-tumoral action and behaves as a potent anti-angiogenic compound for bovine aortic endothelial cells. In this study, we demonstrate anti-angiogenic effects of aeroplysinin-1 on human endothelial cells. Furthermore, the response of angiogenesis related genes to aeroplysinin-1 treatment was studied in human endothelial cells by using gene arrays. The major changes were observed in thrombospondin 1 (TSP-1) and monocyte chemoattractant protein-1 (MCP-1), both of which were down-regulated. These inhibitory effects of aeroplysinin-1 were confirmed by using independent experimental approaches. To have a deeper insight on the anti-inflammatory effects of aeroplysinin-1 in endothelial cells, cytokine arrays were also used. This experimental approach confirmed effects on MCP-1 and TSP-1 and showed down-regulation of several other cytokines. Western blotting experiments confirmed down-regulation of ELTD1 (EGF, latrophilin and seven transmembrane domain-containing protein 1), interleukin 1α and matrix metalloproteinase 1 (MMP-1). These results along with our observation of a dramatic inhibitory effect of aeroplysinin-1 on cyclooxygenase-2 protein expression levels in endothelial cells and a human monocyte cell line suggest that aeroplysinin-1 could be a novel anti-inflammatory compound with potential pharmacological interest.

## Introduction

Aeroplysinin-1 is an 1,2-dihydroarene-1,2-diol produced by Verongida sponges as a chemical defense activated after tissue injury to protect them from invasion of bacterial pathogens [1]–[4]. This compound can be obtained from sponges under controlled in vitro conditions and several analogues have been synthesized [5], [6]. In addition to its antibiotic action [1], [4], [7], aeroplysinin-1 has been shown to have a wide spectrum of anti-tumoral action [8]–[11]. Aeroplysinin-1 has been shown to display a strong anti-tumor effect on EGF-dependent tumor cell lines through its claimed inhibitory effect on the intrinsic protein tyrosine kinase activity of EGF-receptor kinase complex [9].

We have previously characterized aeroplysinin-1 as a potent anti-angiogenic compound *in vitro* and *in vivo* [12]. Most of the *in vitro* assays in that article were carried out with primary cultures of bovine aortic endothelial cells (BAEC). However, although BAEC are widely used as model cell cultures for angiogenesis research, some concerns have been raised due to the facts that they do not come from microvessels and they do not come from humans or model animals [13], [14]. Therefore, a first objective of the present study was to test whether the results obtained in different *in vitro* angiogenesis-related assays are dependent on the origins of the endothelial cells. To fulfil this objective, we have made use of three types of human endothelial cells, namely, EVLC-2 (endothelial venous line cells), RF-24 (an immortalized line of HUVEC, human umbilical vein endothelial cells) and HMEC (immortalized human microvascular endothelial cells). Once demonstrated that our results are consistently reproduced in the three types of tested human endothelial cells, we used primary cultures of HUVEC to evaluate short-term effects of aeroplysinin-1 on angiogenesis-related genes expressed by human umbilical vein endothelial cells (HUVEC), by using commercial angiogenesis gene arrays and alternative validation procedures. Since results point to modulation of genes related with inflammation, we proceeded further by using a commercial cytokine array and alternative validation procedures. Furthermore, several key experiments were also carried out with the THP-1 human monocyte cell line. In this case, results confirmed that monocyte cell proliferation was inhibited and the expression levels of cyclooxygenase-2 protein by these cells was decreased upon treatment with aeroplysinin-1. Altogether, the results shown here support a description of aeroplysinin-1 as an inhibitor of angiogenesis in human endothelial cells and as a new potent inhibitor of pro-inflammatory biomolecules.

## Results

### Aeroplysinin-1 Treatment Inhibits Key Steps of Angiogenesis in Human Endothelial Cells

In a previous report, we identified and characterized aeroplysinin-1 as a potent anti-angiogenic compound affecting several key steps of the process in BAEC [12]. Since that study was carried out using endothelial cells isolated from a great vessel (aorta) from no human source (cow), we began the present study trying to confirm the anti-angiogenic effects of aeroplysinin-1 in human endothelial cells from medium size vessels and microvessels.

Table 1 shows the IC_50_ values determined in proliferation assays using MTT as described in Material and Methods. These values were in the low micromolar range, as it was the case of the published effect on BAEC proliferation [12].

**Table 1:** IC_50_ values for aeroplysinin-1 treatment on human endothelial cells determined by the MTT assay.

Human endothelial cells	IC_50_ (µM)
EVLC-2	3.0±0.3
HMEC	2.6±0.4
RF-24	2.8±0.3
HUVEC	4.7±0.2

**Figure 1:**
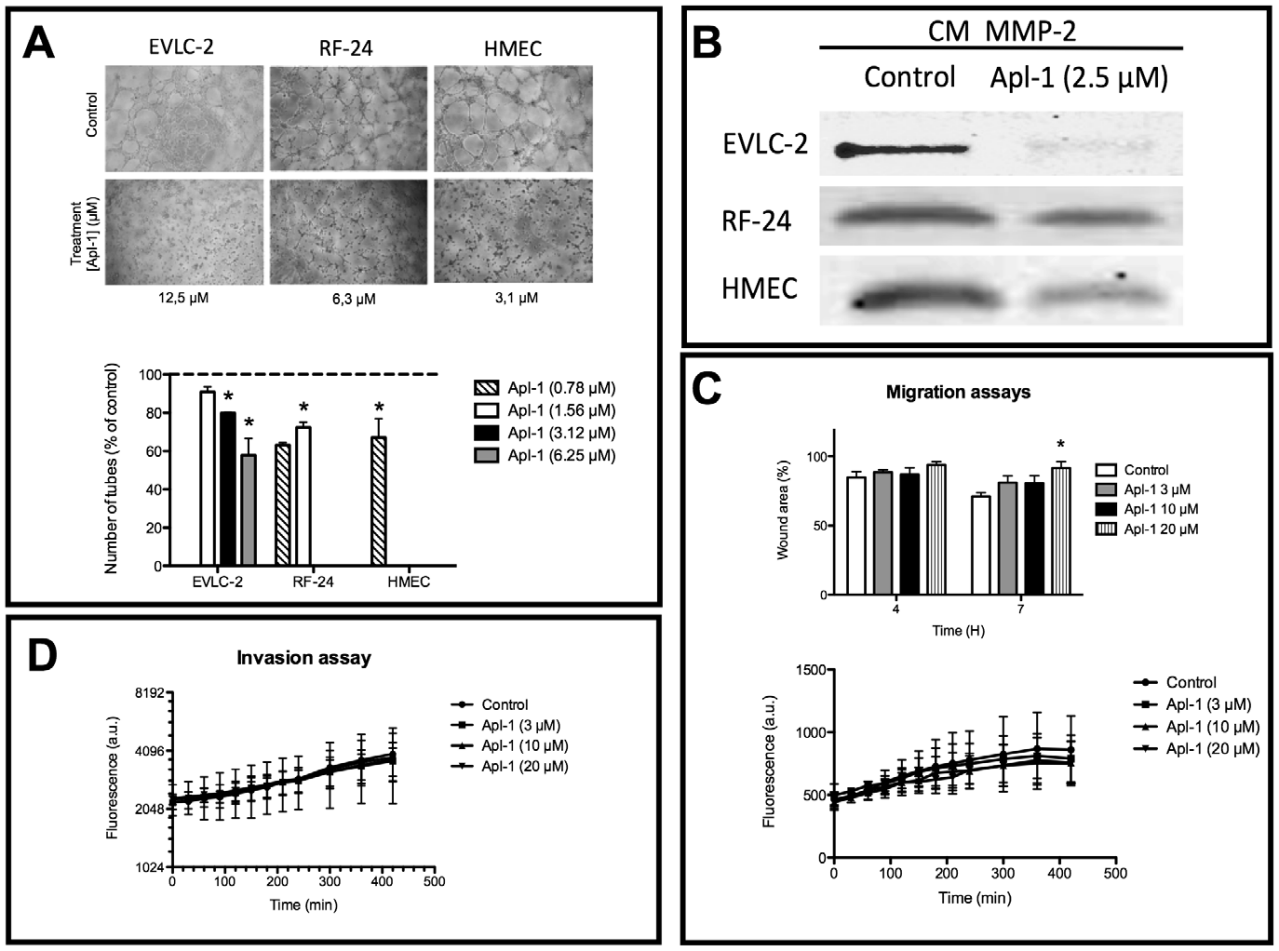
Aeroplysinin-1 inhibits angiogenesis in human endothelial cells. A) Inhibition of the formation of "tubule-like" structures of human immortalized endothelial cells on Matrigel. Representative photographs of control (untreated), and aeroplysinin-1-treated human immortalized endothelial cells on Matrigel after 6 h of treatment. Data are shown on the minimum aeroplysinin-1 concentration yielding total inhibition in each cell line. The histogram shows quantitative analysis of data for the full range of tested endothelial cell types and compound concentrations. B) Aeroplysinin-1 inhibits the expression levels of MMP-2 detected in gelatinolytic assays. The figure corresponds to representative results of gelatinolytic assays showing the levels of MMP-2 activity in control (untreated), and aeroplysinin-1-treated human immortalized endothelial cell conditioned media. C) Aeroplysinin-1 treatment has only a weak inhibitory effect on RF-24 immortalized HUVEC migration in a "wound healing" assay (histogram, above). This effect could not be detected in a semicontinuous fluorescence assay of migration (time course curves, below). In this last case, cells were preloaded with a fluorescence marker and the migration assay was carried out as described in Material and methods. Data are fluorescence measurements of the migrating cells into the lower well at different times of incubation. D) Aeroplysinin-1 treatment has no relevant effect on RF-24 immortalized HUVEC in an invasion assay. Cells were preloaded with a fluorescence marker and the invasion assay was carried out as described in Material and methods. Data are fluorescence measurements of the “invading” cells into the lower well at different times of incubation. All the experiments were carried out as described in Material and methods. Data represent mean±SD for three independent experiments. Symbols indicate significant differences between control-untreated and treated cells (*p<0.01).

The formation of a three-dimensional network of newly formed vessels is the final key event of the angiogenic process. *In vitro*, endothelial cells plated on Matrigel align themselves forming cords (Figure 1A, controls). Figure 1A (treatments) shows that treatment for 6 h with low micromolar concentrations of aeroplysinin-1 resulted in complete inhibition of endothelial cell alignment and cord formation for the three immortalized human endothelial cell lines tested. At lower concentrations of aeroplysinin-1 than those inducing complete inhibition of cord formation, partial inhibition could be observed in a dose-response manner, as shown in Figure 1A (histogram, below).

Matrix metalloproteinase-2 (MMP-2) is a key extracellular enzyme involved in basal membrane remodeling, a required step to allow for endothelial cell migration during active angiogenesis. Figure 1B shows that 2.5 µM aeroplysinin-1 induced a partial inhibition of MMP-2 in conditioned media from RF-24 and HMEC and an almost total inhibition of MMP-2 in conditioned media from EVLC-2.

For the rest of the study, we used either immortalized (RF-24) or primary cultures of HUVEC. Figure 1C (histogram, above) shows that 20 µM aeroplysinin-1 significantly inhibits RF-24 cell migratory potential after 7 h of treatment, as determined in a “wound healing” assay, under conditions not affecting cell viability (data not shown). However, this effect on migration was not detectable when we made use of a migration assay based on a transwell (Figure 1C, time-course curve, below). Figure 1D shows that 3–20 µM aeroplysinin-1 did not significantly affect the RF-24 cell invasive potential through a layer of Matrigel as determined by the invasion assay described in Material and Methods.

### Aeroplysinin-1 Treatment Induces Partial Inhibition of Two Angiogenesis Genes Related to Inflammation in HUVEC

**Figure 2:**
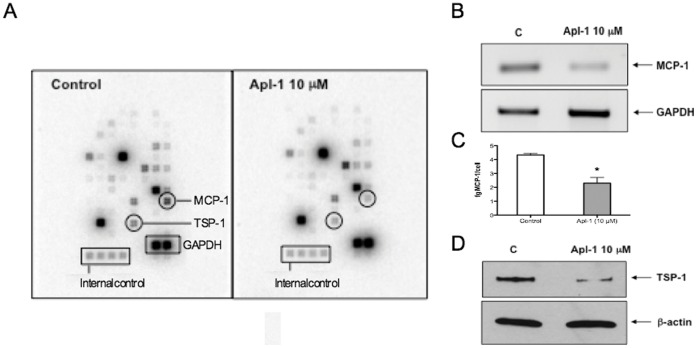
Aeroplysinin-1 decreases the expression levels of MCP-1 and TSP-1 in HUVEC. A) A typical result with GE-Array Q Series Human Angiogenesis Gene Array (SuperArray) is shown. B and C) Validation of the effects of aeroplysinin-1 on the expression levels of MCP-1. MCP-1 messengers were detected by semi-quantitative RT-PCR, using the levels of amplification of GAPDH as a control (B). MCP-1 protein was quantitatively determined by using an ELISA (C). D) Validation of the effects of aeroplysinin-1 on the expression levels of TSP-1. TSP-1 protein levels were detected by Western blotting, using the levels of β-actin as a control. Experiments were carried out as described in the Experimental Section.

In order to evaluate short-term effects of aeroplysinin-1 on angiogenesis-related genes expressed by proliferating HUVEC, we used a human angiogenesis gene array. A typical result is shown in Figure 2A. Due to intrinsic variability of biological samples and the experimental procedure, we only took into account those changes in gene expression consistently repeated in five independent experiments. This stringent requirement was fulfilled by few genes. From them, two genes had the clearest changes upon aeroplysin-1 treatment: thrombospondin 1 (TSP-1) and monocyte chemoattractant protein-1 (MCP-1). Aeroplysinin-1 (10 µM for 6 h) decreased the expression levels of TSP-1 protein and MCP-1 mRNA to 65±8% and 34±13% of their respective control values.

In this study, we confirmed our gene array results by using semi-quantitative RT-PCR for the MCP-1 mRNA expression levels (Figure 2B) and Western blotting for TSP-1 protein levels (Figure 2D). We could not detect a signal for MCP-1 protein in HUVEC by Western blotting due to a lack of sensitivity, but we could observe the inhibitory effect in the level of secreted MCP-1 to the medium by using a high sensitive ELISA assay: in samples of conditioned media from control HUVEC, there was 4.32±0.11 fg MCP-1/cell, whereas in conditioned media from HUVEC treated with aeroplysinin-1 (10 µM for 6 h) there was 2.30±0.41 fg MCP-1/cell (Figure 2C). That means that aeroplysinin-1 treatment decreased the levels of secreted MCP-1 by more than 45%. These additional results undoubtedly show that both MCP-1 mRNA and secreted protein and TSP-1 protein levels are decreased in samples from HUVEC treated with aeroplysinin-1 (10 µM for 6 h), as compared to control, untreated HUVEC.

### Aeroplysinin-1 Treatment Induces Partial Inhibition of the Secretion of Pro-inflammatory Cytokines by HUVEC to Conditioned Media

**Figure 3:**
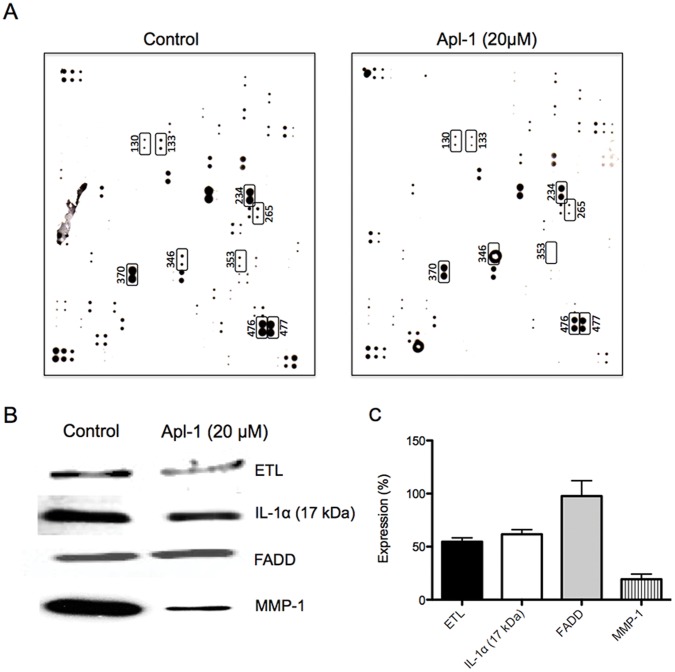
Aeroplysinin-1 decreases the expression levels of MCP-1, TSP-1, ETL, IL-1, FADD and MMP-1, key pro-inflammatory biomolecules in HUVEC. A) A typical result with Human Antibody L-series 507 Cytokine Arrays (RayBiotech) is shown. B) Validation of the effects of aeroplysinin-1 on the expression levels of ETL, IL-1α, FADD and MMP-1. Protein expression levels were detected by Western blotting. C) Quantification of protein levels in aeroplysinin-1-treated samples detected by Western blotting, using the levels of β-actin as a control. Experiments were carried out as described in the Experimental Section. Data are given as percentages of expression, taking the correspondent values of the respective controls as 100%. Means±S.D. for three independent experiments are provided.

To further analyze the anti-inflammatory effects of aeroplysinin-1 in endothelial cells, we determined the cytokines expressed in conditioned media of primary cultures of both control and aeroplysiinin-1-treated HUVEC with the Human Antibody L-series 507 Cytokine Array. Since the levels of inflammatory cytokines released from unstimulated HUVEC are low [15], in these experiments we made use of the SLIGKV-NH_2_ peptide, which behaves as a mild inducer of inflammatory cytokine release from HUVEC [15]. Figure 3A shows a typical result. Due to intrinsic variability of biological samples and the experimental procedure, we only took into account those changes in cytokine levels consistently repeated in three independent experiments. Furthermore, only those changes greater than those corresponding to a factor 2 (that is, an increase of expression of at least the double, or an inhibition of the expression of at least 50%) were considered. These stringent requirements were only fulfilled by a few proteins (16), from which 9 (marked in Figure 3A) were further analyzed. Two of them (marked as cytokines 476 and 477 in the array) corresponded -according to supplier's information- to thrombospondin and thrombospondin-1, respectively. That marked as protein 353 corresponded to MCP-1.

The other 6 proteins marked in Figure 3A were submitted to a validation test by using Western blotting. For two of these proteins, namely, those marked as proteins 265 (interleukin 9) and 346 (low density lipoprotein receptor-related protein 6), Western blotting in our hands provided no signal and they were not further analyzed. Figures 3B and 3C show typical results obtained by Western blotting and the quantification of three independent experiments, respectively, for the rest of the marked proteins 131 (ELTD1), 133 (FADD), 234 (interleukin 1α) and 370 (MMP-1). The decrease in the levels of FADD detected in the array assays could not be confirmed by Western blotting. On the contrary, the effects on the other three proteins could be confirmed. In fact, as Figures 3B and 3C clearly show, aeroplysinin-1 treatment (10 µM for 16 h) of SLIGKV-NH_2_ peptide-stimulated HUVEC produces decreases in the protein expression levels of ELTD1, MMP-1 and IL-1 α in their conditioned media.

### Aeroplysinin-1 Treatment Inhibits Cyclooxygenase-2 Expression in HUVEC

**Figure 4:**
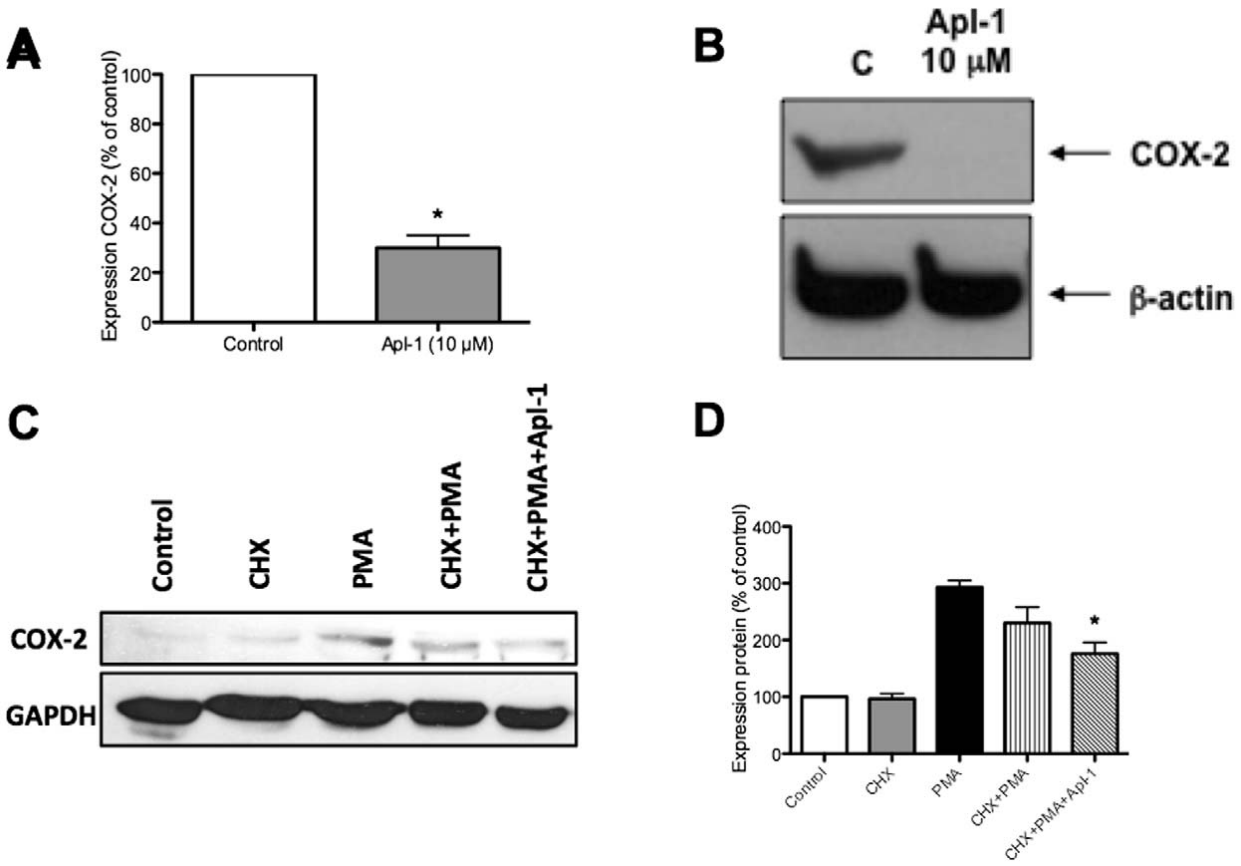
Aeroplysinin-1 abrogates the expression of PMA-induced COX-2 protein. A) PMA (50 ng/mL)-induced COX-2 mRNA was detected by qPCR, using the levels of amplification of GAPDH as a control. B) PMA (50 ng/mL)-induced COX-2 protein levels were detected by Western blotting as described, using the levels of β-actin as a control. C) The expression levels of the protein COX-2 were also analyzed in experiments carried out in the presence of cycloheximide (CHX), as shown in a Western blot. Cells were treated with cycloheximide (90 µg/mL) for 1 h. After washing both control and CHX-pretreated cells were treated in complete medium with PMA (50 ng/mL) in the presence or absence of aeroplysinin-1 (10 µM) for 4.5 h. D) The histogram shows the quantification of three independent experiments as that shown in C.

Since all the previous data clearly stressed the potential of the anti-angiogenic compound aeroplysinin-1 as an anti-inflammatory agent, we wanted to test whether it could down-regulate some other key pro-inflammatory gene not contained in the commercial arrays used. We decided to study COX-2, which is overexpressed in many tumors and plays a key role in atherosclerosis [16]–[18]. Constitutive expression of COX-2 is undetectable in HUVEC. However, COX-2 messenger and protein expression levels in HUVEC are easily induced by phorbol myristate acetate (PMA). Quantitative RT-PCR assay shows that aeroplysinin-1 treatment (10 µM for 4.5 h) decreased the expression levels of PMA (50 ng/mL)-induced COX-2 mRNA by more than 70% (Figure 4A). On the other hand, Western blot assays show that aeroplysinin-1 treatment (10 µM for 4.5 h) completely inhibited PMA-induced expression of endothelial cell COX-2 protein (Figure 4B). Therefore, aeroplysinin-1 seems to have inhibitory effects on PMA-induced expression of COX-2 protein that may involve effects at the transcriptional and post-transcriptional levels. The effect of aeroplysinin-1 on COX-2 protein expression levels was also shown in experiments with cycloheximide (90 µg/mL) pre-treatment for 1 h (Figures 4C and D).

### Aeroplysinin-1 Treatment Inhibits Key Processes and Decreases the Expression Levels of Key Biomolecules in Human Pro-Inflammatory THP-1 Monocytes

**Figure 5:**
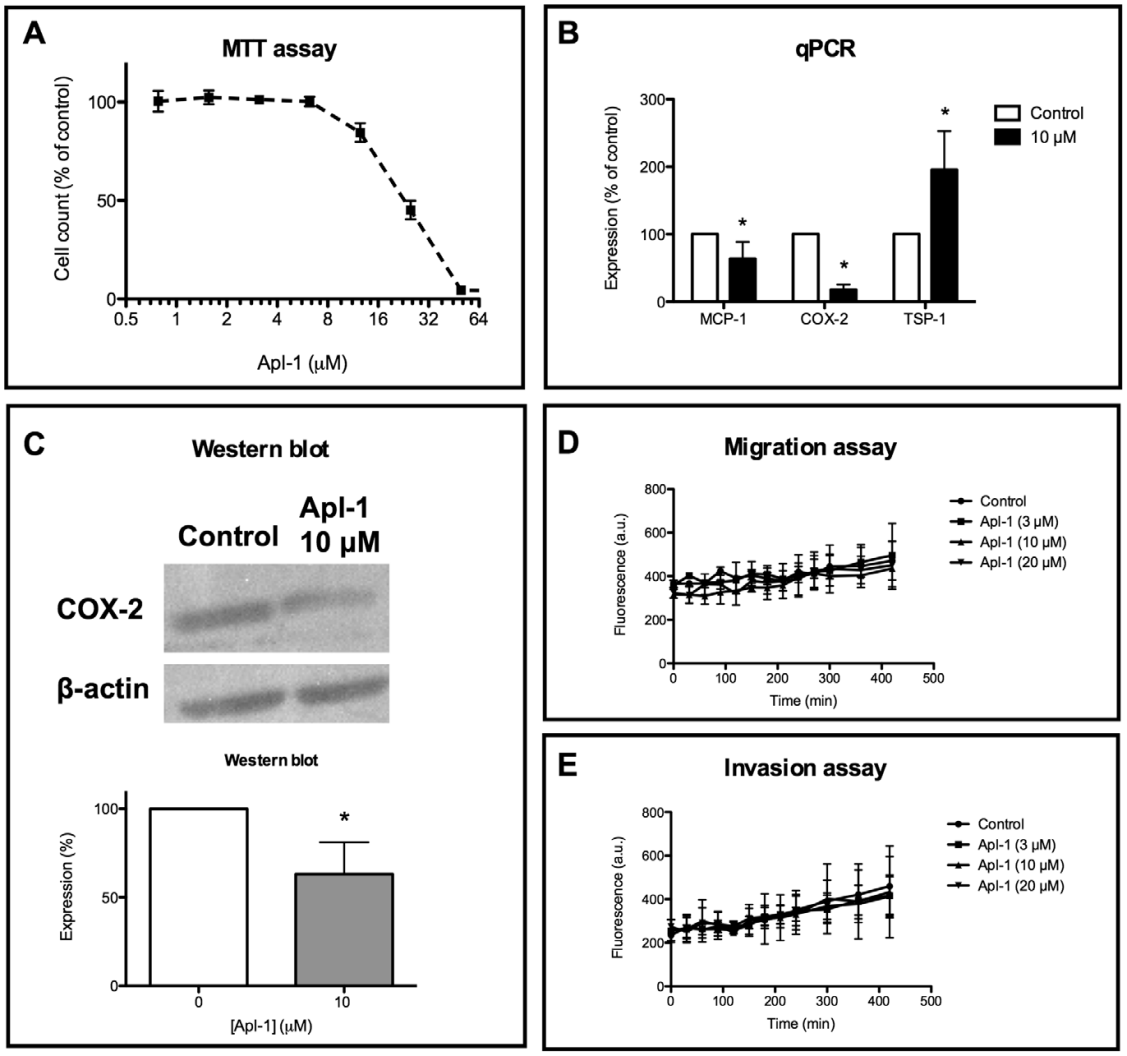
Aeroplysinin-1 inhibits key processes and the expression levels of key biomolecules in THP-1 human inflammatory monocytes. A) Aeroplysinin-1 inhibits THP-1 proliferation as determined by the MTT method. B) Aeroplysinin-1 significantly decreases the expression levels of MCP-1 and COX-2 mRNA as determined by qPCR. C) Aeroplysinin-1 significantly decreases the expression levels of COX-2 protein as determined by Western blot. D) Aeroplysinin-1 has no effect on THP-1 migration. E) Aeroplysinin-1 has no effect on THP-1 invasion. Experiments were carried out as described in Material and methods. Data represent mean±SD for three independent experiments (each one with four replicates of each tested concentration).

Due to the clear inhibitory effect of aeroplysinin-1 on pro-inflammatory biomolecules, we wanted to test the effects of this compound on pro-inflammatory cells. Figure 5 shows that, in fact, aeroplysinin-1 inhibits processes and molecules in THP-1 human pro-inflammatory monocytes. Figure 5A shows that aeroplysinin-1 inhibits THP-1 cell proliferation in a dose-response manner with the IC_50_ value in the micromolar range (24.6±1.0 µM). Figure 5B shows that both MCP-1 and COX-2 mRNA expression levels are significantly reduced with 10 µM aeroplysinin-1 treatment. In contrast, 10 µM aeroplysinin-1 treatment seemd to increase the expression levels of TSP-1 (Figure 5B). Figure 5C shows that the decrease in the expression of COX-2 is also detectable in the protein levels, as shown in Western blot. On the other hand, Figures 5D and E show that aeroplysinin-1 treatment in the 3–20 µM concentration range does not affect migration and invasion capabilities of THP-1 cells.

## Discussion

Our group demonstrated unambiguously that aeroplysinin-1, a brominated compound produced by marine sponges as a mechanism of defense [3], [4], is a potent natural inhibitor of angiogenesis [12]. In that study, most of the in vitro experiments were carried out using bovine aorta endothelial cells (BAEC). As mentioned in the Introduction, BAEC are very frequently used as model cell cultures for angiogenesis research, but some concerns have been raised due to the facts that they do not come from microvessels and they do not come from humans or model animals [13], [14]. In the first part of the present study, our aim was to test whether the in vitro effects of aeroplysinin-1 we observed in BAEC could be reproduced in human endothelial cells obtained from blood vessels with different sizes. The results obtained in the present study clearly reproduced the inhibitory effects of aeroplysinin-1 on endothelial cell proliferation, differentiation and MMP-2 expression (Table 1 and Figure 1) in the same range of micromolar concentrations previously used in BAEC [12] and in studies describing the anti-angiogenic effect of other natural compounds [19], [20]. Only the described slight inhibitory effect of aeroplysinin-1 in the invasion assay when we used BAEC [12] could not be reproduced in the human endothelial cells tested in the present study (Figure 1D). On the other hand, the set of results involving endothelial cell proliferation, differentiation and MMP-2 expression were consistently reproduced in the different human endothelial cells tested, irrespective of their origins. These results give support to the claim that any of these human endothelial cell types can be a useful and valuable model for *in vitro* angiogenesis research.

In our previously published work describing aeroplysinin-1 as a potent anti-angiogenic compound, we identified two molecular targets for its effects, namely, MMP-2 and urokinase [12]. The present study confirms MMP-2 as a molecular target of aeroplysinin-1 (Figure 1B).

A second experimental approach to get more information on potential new molecular targets of aeroplysinin-1 has been the use of commercial low-density arrays of genes related with angiogenesis. In many published articles, authors make only an array experiment or a duplicate experiment, and in only a few published articles these experiments are independently repeated three or more times. Nonetheless, in the present study we only selected those modulatory effects that consistently were repeated in the whole set of five independent experiments. Among those genes fulfilling this strong requirement, the only two ones with decreases higher than 25% in their expression levels were TSP-1 and MCP-1. Gene expression results obtained with gene arrays should be validated by using alternative, independent experimental approaches. Our results in the array experiments were confirmed by different, independent experimental approaches (Figure 2) showing that aeroplysinin-1 treatment down-regulates TSP-1 and MCP-1, two gene products involved in angiogenesis and also related to inflammation. TSP-1 has been described as an endogenous anti-angiogenic compound. However, TSP-1 is multifunctional, playing different biological activities in different cell types [21]. In fact, TSP-1 is one of the major proteins released from activated platelets [22]. Furthermore, TSP-1 expression is up-regulated in inflammation and inflammation-dependent pathologies, including atherosclerosis and coronary artery disease [22]–[24]. Very recently, it has been suggested that some of the pathophysiological connections of TSP-1 expression might be through its function as a hub in a mechanism for autocrine regulation of T cell adhesion and migration [24]. On the other hand, MCP-1 is a member of the C-C class of the beta chemokine family with inflammatory properties [25]. MCP-1 up-regulation is related to macrophage recruitment, angiogenesis and survival in human breast cancer [26]. In addition, MCP-1 plays a critical role in the recruitment and activation of monocytes and in the development of atherosclerosis [27]. We have recently shown that another anti-angiogenic natural compound (namely, epigallocatechin 3-gallate) exerts also a potent inhibitory effect on MCP-1 in inflammatory cells [28], [29]. In conclusion, the extreme selectivity of the conditions established in our experimental setup has been essential to identify two true positives as new molecular targets for the biological effects of aeroplysinin-1. Furthermore, since these are key regulators of inflammation, these results were essential for our decission to devote the last part of the present study to test further the potential of aeroplysinin-1 as a negative modulator of pro-inflammatory biomolecules.

Since both MCP-1 and TSP-1 are pro-inflammatory molecules, data shown in Figure 2 clearly indicated that aeroplysinin-1 behaves as a potent anti-inflammatory agent. To get a deeper insight on the anti-inflammatory effects of aeroplysinin-1 in endothelial cells, we decided to use the Human Antibody L-series 507 Cytokine Arrays, which allows for the simultaneous semiquantitative determination of 507 cytokines in conditioned media of cultured cells. Since the levels of inflammatory cytokines released from unstimulated HUVEC are low [15], in these experiments we made use of the SLIGKV-NH_2_ peptide, a selective agonist of the proteinase-activated receptor-2 (PAR2) [30], [31] that behaves as a mild inducer of inflammatory cytokine release from HUVEC [15].

Expression results obtained with this kind of arrays should be validated by using alternative, independent experimental approaches. This array assay is an additional and independent experimental approach that validates the original observation of the inhibitory effect of aeroplysinin-1 on both TSP-1 and MCP-1 (cytokines 476, 477 and 353 in Figure 3A) expression by HUVEC. Other three new molecular targets of aeroplysinin-1 were selected with the cytokine array assay and confirmed with independent experimental approaches (Figure 3). ELTD1 (EGF, latrophilin and seven transmembrane domain-containing protein 1) is a poorly characterized protein belonging to the secretin family of G-protein-couple peptide hormone receptors that has been previously documented as expressed in heart, lung and vessels [32]. Little is known concerning its functions, although it has been shown to be developmentally regulated in the heart, to be associated with tick burden in cattle, and to be one of eight neuronal genes responsible for subcutaneous fat thickness [32]–[34]. The matrix metalloproteinase-1 (MMP-1), also known as interstitial collagenase, is the only enzyme able to initiate the breakdown of the interstitial collagens (types I, II and III). MMP-1 is involved in the pathogenesis of inflammatory diseases and is upregulated by inflammation [35]–[37]. Finally, interleukin 1α has been described to play a key role in inflammatory and immune responses [38]–[41]. It is important to take these results related with cytokines with caution since, under the conditions used, an effect of aeroplysinin-1 on PAR-2 signaling cannot be ruled out.

Since these results clearly stressed that the anti-angiogenic compound aeroplysinin-1 could also be a potential anti-inflammatory agent, we wanted to test whether it could down-regulate some other key pro-inflammatory gene not contained in the commercial arrays used. We decided to study COX-2, which is overexpressed in many tumors and plays a key role in atherosclerosis [16]–[18]. Our results clearly show that aeroplysin-1 completely abrogates the PMA-induced expression of COX-2 protein with a partial effect on the expression levels of COX-2 mRNA (Figure 4A and B). We could find that pre-treatment with CHX was not able to preclude PMA induction of COX-2 protein expression after 4.5 h of treatment (Figure 4C and D). However, the inhibitory effect of aeroplysinin-1 on this induction was smaller (Figure 4C and D) than that observed on cells not pretreated with CHX (Figure 4B). These data seem to indicate that aeroplysinin-1 could have effects not only on the expression of COX-2 but also on its stability. These results are not conclusive and more experimental effort should be required to address this issue.

Finally, we have studied some of the effects that aeroplysinin-1 treatment can induce in human THP-1 pro-inflammatory monocytes. In fact, we have shown that aeroplysinin-1 inhibits THP-1 cell proliferation in a dose-response manner, although the IC_50_ value is achieved at 8-fold concentrations those required for getting a 50% of inhibition of human endothelial cell proliferation under the assay conditions used in the present study (Figure 5A). We have previously shown that these pro-inflammatory cells are also targets for other anti-angiogenic compounds [29]. Further studies on the effects of aeroplysinin-1 on THP-1 cells shown in the present work include the significant inhibition of the levels of MCP-1 and COX-2 mRNA levels (Figure 5B), the significant decrease in the expression levels of COX-2 protein (Figure 5C) and the lack of effects of aeroplysinin-1 on the migratory and invasive potential of THP-1 cells (Figures 5D and E). Further investigations of the effects of aeroplysinin-1 on inflammatory cells seem warranted.

In conclusion, the results of this study confirm that aeroplysinin-1 inhibits human endothelial cell angiogenesis and suggest that aeroplysinin-1 could be a novel potential anti-inflammatory compound. These results open new ways to the potential pharmacological action of aeroplysinin-1 not only on angiogenesis and cancer [8]–[12], but also on atherosclerosis and inflammation-dependent diseases. Undoubtedly, this suggestion deserves to be studied in the near future.

## Materials and Methods

### Ethics Statement

Primary cultures of HUVEC were obtained from umbilical cords donated at the Maternity of the University Clinical Hospital (Málaga) with the verbal informed consent of donors according to the procedure approved by the ethics committee. All personal data were maintained anonymous and the whole procedure remained anonymous to the authors of this article, who received the donated umbilical cords outside of the operating room. All the procedures were carried out following the rules provided by the bioethical committee of the University of Málaga. This study is part of a research project approved by the bioethical committee of the University of Málaga.

### Chemicals and Reagents

Aeroplysinin-1 was provided by Instituto Biomar (León, Spain) and was dissolved in DMSO and stored at -20°C. Mouse anti-Cox-2 antibody was purchased from Santa Cruz Biotechnology and diluted 1∶1000 in TBS-T containing 1% BSA and 0.02% sodium azide. Mouse anti-TSP-1 antibody was purchased from Neomarkers and diluted 1∶1000 in TBS-T containing 5% non-fat dry milk. The rest of the antibodies used in this study were provided by Abcam. All other reagents, including the peptide SLIGKV-NH_2_, were supplied by Sigma-Aldrich.

### Cell Culture

The three immortalized human endothelial cell lines were kindly supplied by Dr. Arjan W. Griffioen (Maastrich University, Netherlands), who previously characterized them elsewhere [42]. They were grown in RPMI-1640 medium supplemented with glutamine (2 mM), penicillin (50 IU/mL), streptomycin (50 mg/l), amphoterycin (1.25 mg/L), 10% fetal bovine serum and 10% human serum. Primary cultures of human umbilical vein endothelial cells (HUVEC) were isolated from umbilical cords by collagenase digestion [43] and cultured as described elsewhere [12].U937 and THP-1 human inflammatory (monocytes) cells were supplied by ATCC and maintained as recommended by the supplier.

### *In vitro* Angiogenesis Assays

All the in vitro angiogenesis assays used in this work have been extensively described by us elsewhere [12]–[19]. For the MTT proliferation assay, endothelial cells (2.5×10^3^ cells in a total volume of 100 µL of complete medium) were incubated in each well with serial dilutions of aeroplysinin-1. After 3 days of incubation in the dark (37°C, 5% CO2 in a humid atmosphere), 10 µL of MTT (5 mg/mL in PBS) was added to each well and the plate was incubated for further 4 h (37°C). The resulting formazan was dissolved in 150 µL of 0.04 N HCl-2 propanol and read at 550 nm. All determinations were carried out in quadruplicate and at least three independent experiments were carried out. IC_50_ values were calculated as those concentrations of compound yielding 50% cell survival, taking the values obtained for control as 100%.

The rest of the in vitro assays used in this study were carried out under conditions (aeroplysinin-1 concentrations and duration of treatments) that did produce no cytotoxic effect on cells, as determined by modified MTT survival assays.

In the assay for tube formation on Matrigel by endothelial cells, Matrigel (50 µL of about 10.5 mg/mL) at 4°C was used to coat each well of a 96-well plate and allowed to polymerize at 37°C for a minimum of 30 min. 5×104 cells were added with 200 µL of medium. Finally, different amounts of aeroplysinin-1 were added and incubated at 37°C in a humidified chamber with 5% CO_2_ for 6 h. After incubation, cultures were observed and photographed with a NIKON inverted microscope DIAPHOT-TMD (NIKON Corp., Tokyo, Japan). Each concentration was tested in triplicate. “Tubular” structures were counted using the NIH Image 1.6 software.

MMP-2 expression of control and treated human immortalized HUVEC was analyzed by gelatin zymography as previously described by us [19].

The migratory activity of endothelial cells was assessed using a “wound-healing” migration assay. Confluent monolayers in 6-well plates were “wounded” with pipet tips. After washing, cells were supplied with 1.5 mL complete medium in the absence (controls) or presence of different concentrations of aeroplysinin-1. Wounded areas were photographed at different times of incubation. An additional assay of migration is that using a modified Boyden chamber. Migration of fluorescence-labeled endothelial cells was assayed by using a 24-well fluorescence- opaque membrane insert. This assay allows monitoring of the process in real time, because it eliminates the need to remove nonmigratory cells before quantification of migratory cells. Cells were labeled in situ with 5 µg/ml Calcein-AM in complete culture medium for 2 h at 37°C. Cells were added to 8 µm FALCON HTS FluoroBlok inserts (Becton Dickinson) at a density of 5×10^4^ cells/insert in the absence or the presence of the indicated concentrations of aeroplysinin-1. Complete culture medium with FBS was used as a chemoattractant in the lower wells. The inserts were incubated at 37°C, and the real- time kinetics of cell migration was determined by taking readings at the indicated time points. Fluorescence of cells that had migrated through the inserts was measured via the Fluorescence Microplate Reader (FL600FA, BIO-TEK Instruments, Winooski, VT) in the bottom-read mode, with excitation/emission wavelengths of 485/530 nm and a gain setting of 75. The number of cells that migrated through the insert was calculated by interpolation via a standard curve comparing relative fluorescence units with the cell number.

The invasion assay ins a modification of the previously described migration assay. In this case, filters were coated with Matrigel (25 µg/filter).

### Gene Array Assays

For DNA array assays and validation of relevant movements in these arrays, HUVEC were treated with 10 µM aeroplysinin-1 for 6 h. For COX-2 detection, HUVEC were co-treated with PMA 50 ng/mL and 10 µM aeroplysinin-1 for 4.5 h. Control, untreated HUVEC were analysed in parallel for all the cases. Total RNA from control treated HUVEC was isolated in accordance to the protocol provided with the GenElute Mammalian Total RNA Miniprep Kit (Sigma-Aldrich). ^32^P-labeled cDNA, synthesized with the GE-Array AmpoLabeling-LPR Kit (Superarray), was used for screenings of GE-Array Q Series Human Angiogenesis Gene Array (SuperArray). Data were collected and analysed using a Phosphoimager FujiBass 1500 (Fujifilm).

### Cytokine Array Assays

For cytokine array assays and validation of relevant movements in these arrays, 80–85% confluent HUVEC in the absence (control) or presence of 10 µM aeroplysinin-1 were incubated in fresh culture medium supplemented with 100 µM SLIGKV-NH_2_ peptide in the absence of serum for 16 h. Afterwards, conditioned media were collected. Human Antibody L-series 507 Cytokine Arrays (Ray-Biotech) were used to analyse cytokine levels in HUVEC conditioned media following supplier's instructions.

### Quantitative RT-PCR

For quantitative RT-PCR (qPCR), total RNA isolation and complementary DNA synthesis were performed as described above and PCR reactions were done using KAPA SYBR Fast Master Mix (2x) Universal (KAPA Biosystems) in a Eco™ Real-Time PCR System. qPCR was performed in triplicate for each sample according to the manufacturer’s instructions. All qPCR data were normalized to GAPDH expression. Primers, amplicon size, number of cycles and qPCR conditions for each gene are shown in Table 2.

**Table 2:** Primers, amplicon sizes and qPCR conditions.

Gene	Primers	Amplicon size	Number of cycles	PCR conditions
MCP-1	Forward 5′-GCC TTA AGT AAT GTT AAT TCT TATBackward 5′-GGT GTA ATA GTT ACA AAA TAT TCA	241 bp	40	95°/5 min; (95°/1 min;51°/30 s; 55°/30 s)
COX-2	Forward 5′-CTG TAT CCC GCC CTG CTG GTGBackward 5′-ACT TGC GTT GAT GGT GGC TGT CT	282 bp	40	95°/5 min; (95°/1 min;60°/30 s; 55°/30 s)
TSP-1	Forward 5′-TTG TCT TTG GAA CCA CAC CABackward 5′- CTG GAC AGC TCA TCA CAG GA	187 bp	40	95°/5 min; (95°/45 s;60°/30 s; 55°/30 s)
GAPDH	Forward 5′-GGC AAG TTC AAC GGC ACA GTBackward 5′-GCC AGT AGA CTC CAC GAC AT	157 bp	40	95°/5 min; (95°/45 s;60°/30 s; 55°/30 s)

### Western Blotting

For Western blotting, treated and control cells were lysed in Laemmli’s loading buffer 2X and boiled for 5 min at 95°C; samples were separated in a SDS-PAGE electrophoresis and blotted onto a nitrocellulose membrane. After blocking in TBS-T plus 5% w/v dry non-fat milk, membranes were probed with primary antibodies overnight at 4°C. Membranes were washed in TBS-T and probed with secondary antibody linked to horseradish peroxidase for 1 hour at room temperature. After washing, membranes were developed using the ECL™ system (Amersham Biosciences) or the SuperSignal West Pico Chemiluminiscent Substrate system (Thermo). For validation of relevant movements in cytokine array assays, Western blotting was carried out exactly as indicated in the instructions for users of this array.

### Determination of MCP-1 Secretion to HUVEC Culture Supernatants

MCP-1 protein in HUVEC culture supernatants after the treatment with aeroplysinin-1 was measured using a commercial MCP-1 Human Biotrak Easy ELISA kit (GE Healthcare) according to the manufactureŕs instructions. The absorbance at 450 nm was determined using a microplate reader 680 (Bio-Rad). The MCP-1 protein levels in the conditioned medium were carried out in four independent cultures.

### Statistical Analysis

For all the assays, at least three independent experiments were carried out. Results are expressed as mean+S.D. Statistical significance was determined by the Students paired sample test. Values of p<0.05 were considered to be significant.
